# Simple Method for Controlling Epistaxis

**Published:** 1891-02

**Authors:** 


ARTICLE VIII.

### SIMPLE METHOD FOR CONTROLLING EPISTAXIS.

Dr. W. W. Parker, of Richmond, Va., says, in the New York *Medical Record*: The plan of arresting hemorrhage from the nose, which I here describe, I have used for thirty years without one failure. When I first began to practice I used Bellocq's instrument, but I found it painful, and, in small children, exceedingly troublesome of application. Two months ago a man twenty-three years old, in this city, was operated upon for irregular septum (a small part of the bone was removed), which was followed by troublesome hemorrhage. The operation was by a very skillful specialist, who, upon application of the patent the

---

‖ Lydston, "Jour. Amer. Med. Assoc., 1886, vi., p. 654.

day after the operation, plugged the posterior nares with difficulty. But the hemorrhage persisted for three days, when the patient, whose father's family I had attended for many years, applied to me in company with his father. I of course, declined to have anything to do with the case, and urged him to return at once to the specialist who could do everything that was necessary. This he positively refused to do, and said if I would not attend him he would get some one else. He had visited the doctor three times, but the bleeding continued. As it was ten o'clock at night when he came to my office, I told him I would stop the bleeding and would see his doctor next morning and get him to call at the patient's house. This I did. I stopped the bleeding at once, and kept the patient and his father in my office till twelve o'clock at night to see if the hemorrhage really was arrested. This was the last of the bleeding. This man was of a hemorrhagic diathesis, and some three weeks after this, he had hemorrhages from the lungs, which had continued occasionally up to this date. At the Red Sulphur Springs of Virginia he gained twenty pounds but the bleeding from the lungs continued. He is of a phthisical family. I have been thus particular so that I might satisfy the reader that this was a bad case of epistaxis.

My plan is not only effectual, but is easy of application and absolutely painless, and can be probed in the smallest patients. The little device which I use is made of fifteen of the long threads of patent lint, size three and one-half or four inches long, which I double on themselves and tie in the middle, and let one end of the string be six or eight inches long so as to pull the plug out when necessary. When doubled on itself it looks like a "comet" in miniature with a nucleus and thirty tails (or twice the number of threads used). A probe, or soft piece of pine but little bigger than a match, is pressed up against the centre, and it is passed back upon the floor of the nasal cavity and pushed on till you reach the posterior nares. This will be known both by the resistance and the length of the probe, or the depth which you have reached. Then slowly withdraw the probe and plug the anterior nares and you have arrested the bleeding. These twenty or thirty ends floating in the blood at once coagulate it. The passage of the soft lint gives no pain whatever. In persuading children to submit to the operation, I often pass the lint up my own nose to satisfy them it gives no pain. If lint is not at hand I use the largest size spool cotton.

Some years ago I called the attention of one of our local societies to this matter, and it was mentioned in a local journal, but attracted no attention. Seeing the notice of Dr. Fridenberg's method in the *Medical Record*, I send this to you hoping it will have a wider circulation. Any sensible layman can perform this operation. Many years ago I was wont sometimes to remain at the patient's house for hours, fearing a return of the bleeding, but do so no longer. The plug is removed in from twenty-four to forty-eight hours. It gives no pain and the patient is willing for it to remain. The other methods are all painful in execution, and the discomfort, while the plug remains, is very considerable.

